# Effects of Keemun and Dianhong Black Tea in Alleviating Excess Lipid Accumulation in the Liver of Obese Mice: A Comparative Study

**DOI:** 10.3389/fnut.2022.849582

**Published:** 2022-03-15

**Authors:** Wenjing Liao, Suyu Liu, Yunxi Chen, Yashuai Kong, Dongxu Wang, Yijun Wang, Tiejun Ling, Zhongwen Xie, Irada Khalilova, Jinbao Huang

**Affiliations:** ^1^State Key Laboratory of Tea Plant Biology and Utilization, School of Tea and Food Science and Technology, Anhui Agricultural University, Hefei, China; ^2^School of Grain Science and Technology, Jiangsu University of Science and Technology, Zhenjiang, China; ^3^Life Sciences Department, Center for Cell Pathology Research, Khazar University, Baku, Azerbaijan

**Keywords:** black tea, diet-induced obesity, fatty liver, lipid metabolism, fecal excretion

## Abstract

The chemical compositions of black teas differ greatly and may have different health benefits; however, systematic investigations into such benefits are lacking. Here, the chemical profiles of Keemun black tea (KBT) and Dianhong black tea (DBT), two common categories of tea in China, were analyzed, and their lipid-lowering effects in male C57BL/6 mice fed a high-fat diet (60% energy from fat) or the diet supplemented with 2% black tea powder for 15 weeks were investigated. The compounds most crucial in differentiating KBT and DBT were determined to be phenolic compounds, theanine, and D-psicose. DBT was more effective than KBT in preventing excess hepatic fat accumulation. Both black teas effectively and comparably altered the mRNA levels of hepatic lipid–metabolizing genes. DBT had more favorable effects in stimulating fecal fat excretion than did KBT. The differentiating compounds with the higher values of variable importance in the projection (VIP) might predominantly contribute to the different health benefits; however, the most essential compound or combination of compounds requires clarification.

## Graphical Abstract

Schematic diagram of the effects of two black teas in alleviating excess hepatic lipid accumulation.
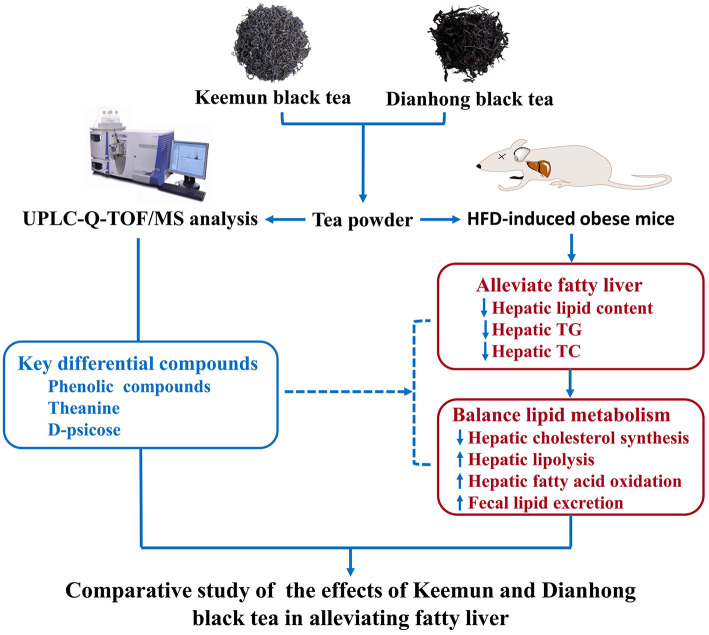

## Introduction

Fatty liver, which is characterized by excessive hepatic lipid storage, is closely related to high-fat diet (HFD)-induced obesity. Numerous pathophysiological mechanisms are involved in the development of obesity, and triglycerides and cholesterol in hepatic and intestinal tissues are crucial factors in the regulation of lipid metabolism and energy balance (1). Dietary control is considered a key strategy in the prevention of fatty liver (2). Natural phytochemicals, such as polyphenols, have demonstrated protective effects against fatty liver (3). Tea is popular worldwide, and its lipid-lowering and weight-reducing effects have been frequently reported in animal studies and human interventions (4).

Black tea is the most-consumed tea beverage in the world, and its health benefits, including anti-obesity anti-atherosclerosis properties and the prevention of fatty liver, have been frequently reported (5). As underlying mechanisms, tea inhibits fat synthesis and promotes fecal lipid excretion and fat oxidative decomposition (6). Black tea contains a large quantity of biologically active substances, such as catechins, theaflavins, thearubigins, theanine, alkaloids, phenolic acids, and tea polysaccharides, which are the material basis of its efficacy. The chemical profiles of black tea categories differ greatly. They are influenced by the plant cultivar, garden environment, fresh leaf maturity, and manufacturing process among other factors (7). The genetic background of the tea plant is a vital. Taxonomically, the cultivated varieties of tea plant are generally classified into two groups: *Camellia sinensis* var. *assamica* and *C. sinensis* var. *sinensis* (8). Assam black tea from India and Dianhong black tea (DBT) from China are representative of the large-leaf cultivar *C. sinensis* var. *assamica*, and Keemun black tea (KBT) from China and Darjeeling black tea from India are produced by the small-leaf cultivar *C. sinensis* var. *sinensis*. Differences in the chemical compositions of black tea categories lead to different health benefits; however, few comparative studies have systematically investigated these differences in chemical profiles and efficacies.

In the present study, the chemical profiles of KBT and DBT and their effects in preventing HFD-induced fatty liver were examined. KBT is grown in Anhui province, China, and possesses unique floral and honey aromas (9). DBT is from Yunnan province, China, and has a higher phenolic content and strong, mellow aromas (10). Ultra performance liquid chromatography-quadrupole-time of flight mass spectrometry (UPLC-Q-TOF-MS/MS) was used to analyze the chemical compositions of KBT and DBT. Male C57BL/6 mice (7 weeks old) were fed different diets: a low-fat diet (LFD) or an HFD with or without black tea powder supplementation. The expression levels of key genes pertaining to lipid metabolism were measured using the PCR technique, and high-throughput sequencing was used to screen gut microbiota changes in fecal samples.

## Materials and Methods

### Tea Samples

Fresh KBT and DBT leaves with the same maturity (one shoot and two young leaves) were picked in May 2019. The sample of KBT was processed and obtained from local tea factory (Qimen, Anhui, China), and DBT was processed and obtained from local tea factory (Yunnan, China). All samples were sealed stored at −20°C before analysis.

### Untargeted Metabolomic Analysis by LC-MS

Non-targeted metabolomic analysis was conducted as reported (11) with minor modifications. An Agilent 1290 liquid chromatography system connected to a time-of-flight mass spectrometer (Agilent, Palo Alto, CA) and an RP18 column (50 × 2.1 mm^2^, 1.7 μm) (Waters, Milford, MA) was used to analyze the samples. The gradient elution, instrument parameters, and metabolomic analysis were as described by Guo et al. (9). Qualitative and quantitative analyses were performed using the mass spectrometric data obtained. In brief, Optimus (Version 1.5.1) was employed to transform the original data obtained from LC-MS/MS. SMICA-P software (V14.1 Umetrics, Umea, Sweden), the Global Natural Products Social Molecular Networking platform, and Cytoscape (version 3.8.0) were used for multifactorial analysis and the identification of compounds. The final quantitative analysis was completed using MassHunter Qualitative Analysis (B.07.00).

### Animals, Diets, and Treatments

The animal experiment was conducted in compliance with institutional animal care guidelines and approved by the Committee of Anhui Agricultural University (approval number AHAU2019025). Forty-eight C57BL/6 mice (male, 6 weeks) were obtained from the Model Animal Research Center of Nanjing University (Nanjing, China). Upon arrival, the mice were maintained in a specific-pathogen-free environment and reared in ventilated cages under controlled conditions (25 ± 2°C, 50 ± 5% relative humidity) with a 12-h light/dark cycle. All the animals had ad libitum access to diet and tap water during the entire rearing experiment.

The experimental groups received the following diets after acclimation for 1 week: (1) LFD (TP2330055BC), (2) HFD (TP2330055B, Supplementary Table 1), (3) HFD and 2% KBT powder (HFKB, HFD containing 2.0% KBT [w/w]), or (4) HFD and 2% DBT powder (HFDB, HFD containing 2.0% DBT [w/w]). The animal feed was provided by a commercial company (Trophic Animal Feed, Nantong, China) and kept under freezing temperature (−20°C).

During the 15-week experiment, the body weight of the mice was measured weekly. Consumption of food and water was recorded every other day. Feces were collected every 2 weeks and stored at −80°C. After 15 weeks of treatment, the animals were sacrificed under anesthesia with chloral hydrate (4%, w/w) by intraperitoneal (i.p.) injection. Blood was collected through cardiopuncture, and serum samples were obtained after centrifugation. Immediately afterward, liver and white adipose tissues were harvested and weighed. The small and large intestines were then collected, cut out, and rinsed in cold 0.9% saline, and the cecal contents were collected at the same time. All the samples were snap-frozen in liquid nitrogen and stored at −80°C for subsequent analyses.

### Analysis of Blood Biochemical Parameters

Commercial kits (Jiancheng Technology, Nanjing, China) were used to determine the levels of serum lipids and aminotransferases. The measurements were conducted in strict accordance with manufacturer instructions.

### Hepatic Histochemical Analysis and Lipid Content Determination

For histochemical analysis, the liver samples were fixed in formalin and embedded in paraffin. Hematoxylin and eosin (H&E) staining was performed in compliance with the standard procedure, and the samples were examined at 200-fold magnification.

Lipids in the liver were extracted and measured according to our previous study (12), and the levels of hepatic triglycerides (TG) and total cholesterol (TC) were determined using the methods used with the serum samples.

### Total Fecal Bile Acid and Lipid Content Analysis

The total bile acids in the feces were extracted and determined according to the method described by Kim et al. (13). A commercial assay kit (Huili Biotech, Changchun, China) was used to determine the concentrations of bile acids extracted. The analysis of lipid content in feces was conducted identically to that for livers.

### Analysis of Gene Expression Through RT-qPCR

The total RNA of liver and small intestine tissues was extracted using an RNA extraction kit (Tiangen, Beijing, China), and reverse transcription was performed with a GoScript reverse transcription system (Promega, Madison, WI), following the manufacturer's instructions. The mRNA levels of hepatic and intestinal genes were measured with a PCR mix kit (Life Technologies, Carlsbad, CA), normalized to the expression level of β-actin, and calculated using the 2^−ΔΔCT^ method. The sequences of genes and primers were obtained and designed using the NCBI Gene Bank database and BLAS tools. The gene ID and primer sequences are presented in Supplementary Table 2.

### Short-Chain Fatty Acids and Gut Microbiota Analysis

The determination of fecal short-chain fatty acids (SCFAs) was done with reference to the method described by Tian et al. (14), with modifications. Briefly, an Agilent 7890A GC system connected to a flame ionization detector and an HP-INNOWAX column (30 m × 0.25 mm × 0.25 μm, Agilent Technologies) was used. The column temperature was regulated as follows: 100°C maintained for 1 min, then an increase of 5°C/min for 16 min to achieve a temperature of 180°C, which was maintained for 4 min.

The fecal genomic DNA was extracted with the TIANamp Stool DNA Kit (Tiangen, Beijing, China) according to the manufacturer's instructions. The total bacterial DNA was sent to Novogene Co., Ltd. (Beijing, China) in dry ice. For high-throughput sequencing, the hypervariable region of the 16S rRNA (V3-V4) was selected for amplification. Sequences with a similarity of 97% according to UPARSE were clustered into operational taxonomic units, which were randomly subsampled (15). Alpha diversity analysis was performed to measure the complexity of species diversity. The species complexity in the samples was evaluated using beta diversity analysis. The alpha and beta diversities of both weighted and UniFrac results were assessed using R software (version 2.15.3 http://www.r-project.org/).

### Statistical Analysis

The data are expressed as mean ± standard error of the mean. The multisample analysis was performed through one-way analysis of variance and Tukey's *post-hoc* test by using IBM SPSS Statistics for Windows, version 22 (IBM Corp., Armonk, NY). A difference was considered significant if *p* < 0.05.

## Results

### Untargeted Metabolomic Analysis of KBT and DBT

**Figure 1:**
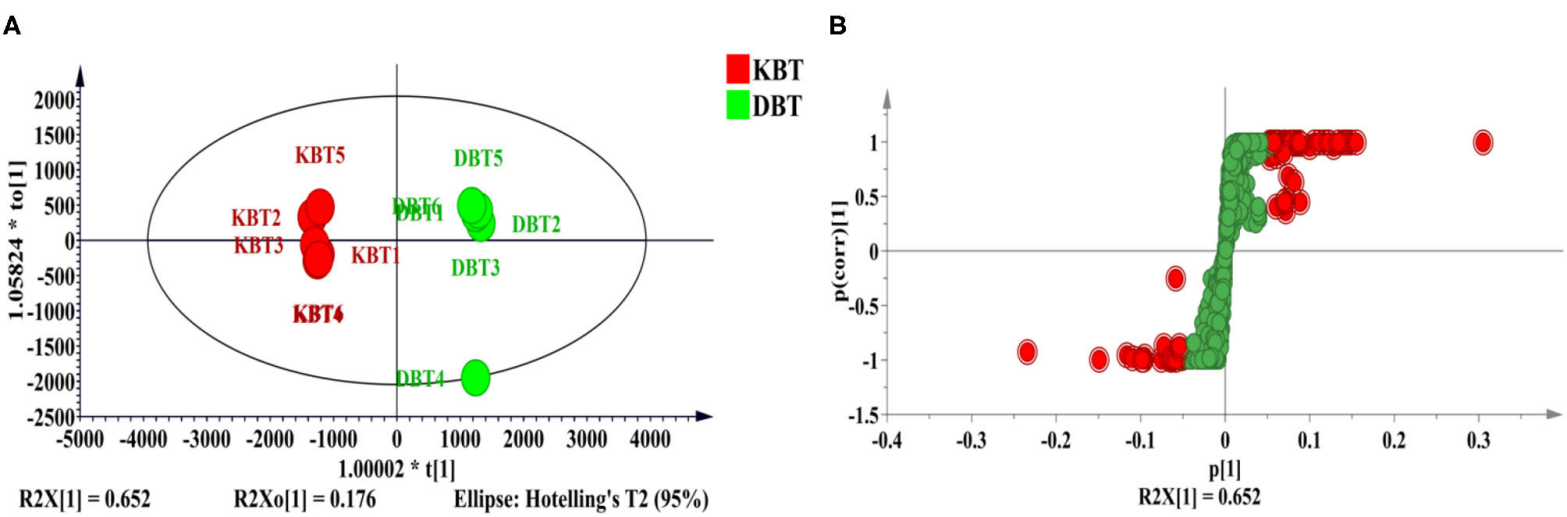
Multivariate analysis of compounds detected in mass spectrometry. Keemun black tea (KBT), Dianhong black tea (DBT). **(A)** OPLS-DA; **(B)** S-plot.

To systematically examine the differences in the chemical profiles of KBT and DBT, UPLC-Q-TOF/MS was employed. Orthogonal partial least squares discriminant analysis (OPLS-DA) provided a clear classification for the two types of black tea (Figure 1A). Compounds with a VIP value ≥ 2 and *p*[1] value in the S-plot > |0.05| were selected as the key differentiating compounds to distinguish KBT and DBT (Figure 1B). A total of 33 critical compounds in negative mode were screened and identified. As summarized in Table 1, three carbohydrates and carbohydrate conjugates, eight phenolic acids and contractive phenolic acids, three catechins, nine flavonoids, one tannin, two hydrolyzable tannins, two amino acids and derivatives, one gallic acid and derivatives, three citric acids or isomers, and one hyperoside were identified. Most of the 10 marker compounds with the highest VIP values were phenolic acids, flavonoids, theanine, hydrolyzable tannins, and D-psicose. A total of 25 compounds of the 33 critical ones were phenolic, indicating their vital roles in distinguishing these two categories of black tea.

**Table 1:** Compounds crucial in differentiating Keemun black tea and Dianhong black tea.

**No**.	**RT (min)**	***m*/*z***	**VIP**	**Identification**	**Classification**
1	0.84	191.059	11.41	Quinic acid or it's isomer	Phenolic acids and contractive phenolic acids
2	7.96	447.099	5.61	Kaempferol-3-O-glucoside or it's isomer	Flavonoids
3	0.5	173.092	5.61	Theanine	Amino acids and derivatives
4	7.69	463.091	5.54	Isoquercitrin or it's isomer	Flavonoids
5	4.32	633.078	5.35	[(1R,21S,23R)-6,7,8,11,12,13,22,23-octahydroxy-3,16-dioxo-2,17,20-trioxatetracyclo[17.3.1.04,9.010,15]tricosa-4,6,8,10,12,14-hexaen-21-yl] 3,4,5-trihydroxybenzoate	Hydrolyzable Tannins
6	7.71	739.214	5.24	Kaempferol-3-O-galactoside-6”-rhamnoside-4”'-rha or it's isomer	Flavonoids
7	3.09	337.098	5.01	3-O-Coumaroylquinic acid	Phenolic acids and contractive phenolic acids
8	8.11	447.098	4.75	Kaempferol-3-O-glucoside or it's isomer	Flavonoids
9	6.49	635.095	4.62	1,2,3-Tri-O-galloyl-beta-D-glucose	Tannins
10	0.54	179.06	4.51	D-Psicose	Carbohydrates and carbohydrate conjugates
11	0.5	145.065	4.35	L-Glutamine	Amino acids and derivatives
12	6.2	457.083	4.17	Epigallocatechin gallate	Catechins
13	1.24	169.018	3.93	Gallic acid	Gallic acid and derivatives
14	0.52	387.12	3.61	Coniferyl alcohol + O-Hex	Carbohydrates and carbohydrate conjugates
15	7.57	463.069	3.31	Isoquercitrin or it's isomer	Flavonoids
16	3.38	337.098	3.31	3-O-Coumaroylquinic acid or it's isomer	Phenolic acids and contractive phenolic acids
17	4.95	337.098	3.28	3-O-Coumaroylquinic acid or it's isomer	Phenolic acids and contractive phenolic acids
18	4.3	337.098	3.18	Coumaroyl quinic acid	Phenolic acids and contractive phenolic acids
19	3.77	353.093	3.01	3-O-Coumaroylquinic acid or it's isomer	Phenolic acids and contractive phenolic acids
20	7.47	609.15	2.89	Rutin or it's isomer	Flavonoids
21	8.16	447.098	2.85	Kaempferol-3-O-glucoside or it's isomer	Flavonoids
22	6.91	457.08	2.85	Epigallocatechin gallate	Catechins
23	0.84	191.023	2.78	Citric acid or it's isomer	Citric acid or it's isomer
24	7.63	463.093	2.62	Hyperoside	Flavonoids
25	7.39	609.152	2.53	Rutin or it's isomer	Flavonoids
26	0.56	191.023	2.5	Citric acid or it's isomer	Citric acid or it's isomer
27	7.35	441.089	2.42	Epicatechin gallate	Catechins
28	0.56	191.06	2.39	Quinic acid or it's isomer	Phenolic acids and contractive phenolic acids
29	0.56	191.024	2.35	Citric acid or it's isomer	Citric acid or it's isomer
30	4.25	633.079	2.17	[(1R,21S,23R)-6,7,8,11,12,13,22,23-octahydroxy-3,16-dioxo-2,17,20-trioxatetracyclo[17.3.1.04,9.010,15]tricosa-4,6,8,10,12,14-hexaen-21-yl] 3,4,5-trihydroxybenzoate	Hydrolyzable Tannins
31	7.62	739.213	2.15	Kaempferol-3-O-galactoside-6”-rhamnoside-3”'-rha or it's isomer	Flavonoids
32	1.02	179.059	2.08	Psicose	Carbohydrates and carbohydrate conjugates
33	4.06	353.092	2	Chlorogenic acid	Phenolic acids and contractive phenolic acids

### Effects of Dietary Black Tea on Visceral Fat Mass and Serum Lipids

**Figure 2:**
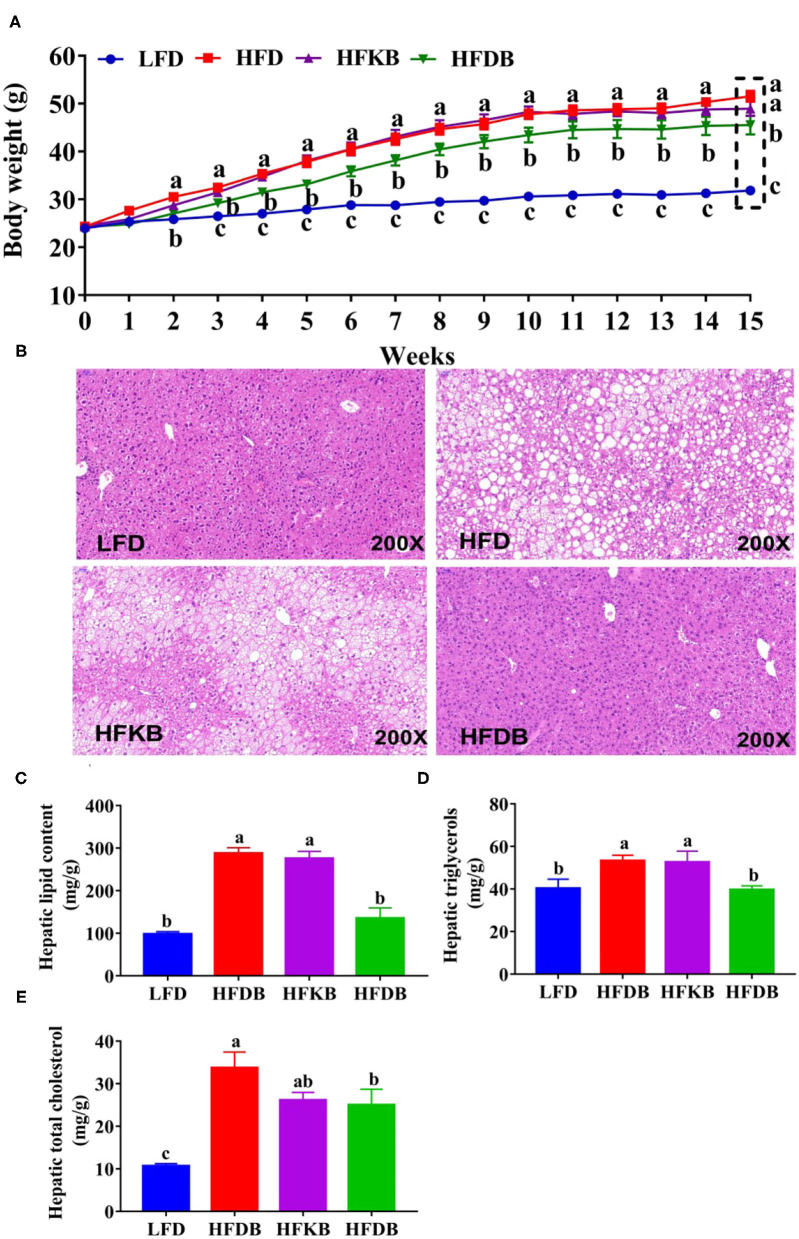
Effects of black tea on body weight and fat accumulation in the liver. LFD, low-fat diet; HFD, high-fat diet; HFKB, HFD + KBT diet (containing 2.0% Keemun black tea); HFDB, HFD + DBT diet (containing 2.0% Dianhong black tea). **(A)** body weight; **(B)** hematoxylin and eosin–stained slices of liver; **(C)** total lipid content in the liver; **(D)** hepatic triglyceride content; **(E)** total cholesterol content in the liver. Data are presented as mean ± SEM (*n* = 12). ^a,b,c^*p* represents significant differences among groups (ANOVA, *p* < 0.05).

UPLC-Q-TOF/MS analysis indicated that the contents of phenolic acids, catechins and flavonoids between KBT and DBT were different. To clarify the differences in the anti-obesity effects between KBT and DBT in HFD-fed mice, the body weight gain and visceral fat mass of the experimental animals were measured. As shown in Figure 2A, no significant differences were observed in the initial body weight of the four groups. After week three, HFD-treated mice had significantly higher body weight than did mice in the LFD group, and they eventually gained 81.4% more weight increase compared with the LFD-treated mice. Dietary DBT significantly decreased body weight by 30.7%. Although the body weight of HFKB-treated mice exhibited a decreasing trend compared with HFD mice at week 15, no significant difference was observed (food consumption of the four groups was comparable throughout the experimental period; Supplementary Figure 1). The HFD notably increased visceral fat mass, and HFKB and HFDB treatments significantly decreased perirenal adipose mass deposition by 22.8 and 32.8%, respectively (Table 2). No significant differences in mesenteric and epididymal adipose mass among the HFD, HFKB, and HFDB groups were noted (Table 2). Similarly, the HFD significantly enhanced the levels of serum lipid parameters (LDL-C, HDL-C, and TC), and black tea treatments failed to prevent these increases (Supplementary Table 3). Moreover, the serum TG levels of mice in the four groups were comparable.

**Table 2:** Liver and visceral fat mass and concentrations of serum AST and ALT.

	**Percentage of body weight (%)**			
	**LFD**	**HFD**	**HFKB**	**HFDB**
Perirenal adipose	0.82 ± 0.29^c^	3.41 ± 0.47^a^	2.82 ± 0.68^b^	2.56 ± 0.71^b^
Mesenteric adipose	1.20 ± 0.24^b^	3.08 ± 0.56^a^	2.84 ± 0.93^a^	2.80 ± 0.96^a^
Epididymal adipose	2.70 ± 0.77^b^	4.70 ± 0.86^a^	4.32 ± 1.34^a^	4.99 ± 0.85^a^
Liver	4.02 ± 0.67^b^	7.00 ± 1.67^a^	6.97 ± 1.71^a^	6.12 ± 1.24^a^
AST (U/L)	29.11 ± 2.91	24.24 ± 3.32	19.39 ± 4.65	27.23 ± 5.26
ALT (U/L)	5.17 ± 1.89^c^	50.67 ± 16.06^a^	35.99 ± 9.43^b^	32.04 ± 8.13^b^

*AST, aspartate aminotransferase; ALT, alanine aminotransferase. Values are expressed as mean ± SEM (n = 12). Different letters indicate significant differences (ANOVA, p < 0.05)*.

### Effects of Dietary Black Tea on the Development of Fatty Liver

As shown in Table 2, the HFD significantly increased liver weight, and black tea treatments slightly but not significantly decreased the ratio of total liver weight to body weight. The levels of serum ALT in the mice in the HFD group were significantly (8.8-fold) higher than those in the LFD group, and HFKB and HFDB treatments significantly decreased this parameter by 32.3 and 40.9%, respectively (Table 2).

We also conducted liver histopathological analysis using slices stained with H&E. No sign of fatty liver was observed in the LFD mice (Figure 2B), and the hepatocytes of these samples were morphologically intact, with clear borders, and neatly arranged. In the HFD groups, numerous large fat vacuoles were observed, with the nucleus moving to one side and no clear boundary between cells (Figure 2B). Black tea treatments effectively prevented the development of HFD-induced fatty liver, especially in the HFDB-treated mice, whose liver slices were similar in appearance to those of LFD mice. Furthermore, total hepatic lipid, TG, and TC levels were significantly increased in the HFD groups relative to the LFD group (Figures 2C–E). Similar to the implications of the histopathological data, DBT treatment completely prevented the HFD-induced increase of hepatic total lipids and TG. DBT treatment also significantly decreased hepatic TC content by 37.9%. KBT treatment slightly but not significantly prevented lipid accumulation in the livers of experimental mice.

### Effects of Dietary Black Tea on Total Bile Acid and Lipids in Fecal Samples

**Figure 3:**
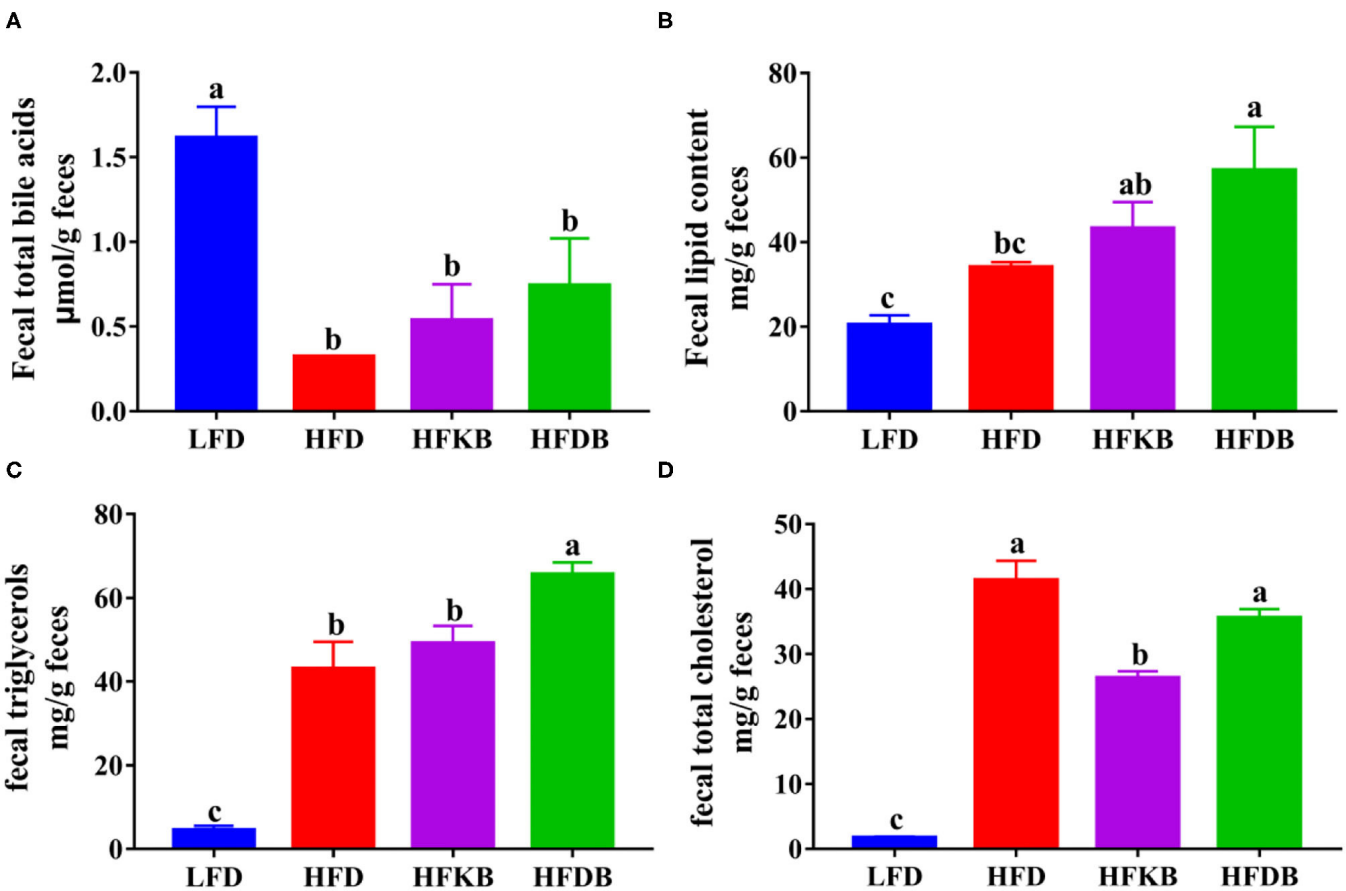
Effects of black tea on fecal total bile acids and lipids. **(A)** fecal total bile acids; **(B)** fecal total lipids; **(C)** fecal triglycerides; **(D)** fecal total cholesterol. Data are presented as mean ± SEM (*n* = 12). ^a,b,c^*p* represents significant differences among groups (ANOVA, *p* < 0.05).

To examine the effects of dietary black tea on intestinal fat absorption, we measured total bile acid and lipid levels in murine feces. The level of fecal total bile acids in the HFD groups was significantly lower than that of the LFD group. HFKB and HFDB slightly increased the fecal excretion of bile acids; however, no significant differences were observed (Figure 3A). Higher levels of fecal total lipids, TGs, and cholesterol were also observed in mice in the HFD group. Mice who underwent DBT treatment exhibited higher excretion of fecal lipids and TGs compared with the mice in the HFD group (Figures 3B–D). Similar to the aforementioned parameters, the fecal lipid–promoting effects of KBT were lower than those of DBT.

### Effects of Dietary Black Tea on Lipid-Metabolizing Gene Expression in the Liver

**Figure 4:**
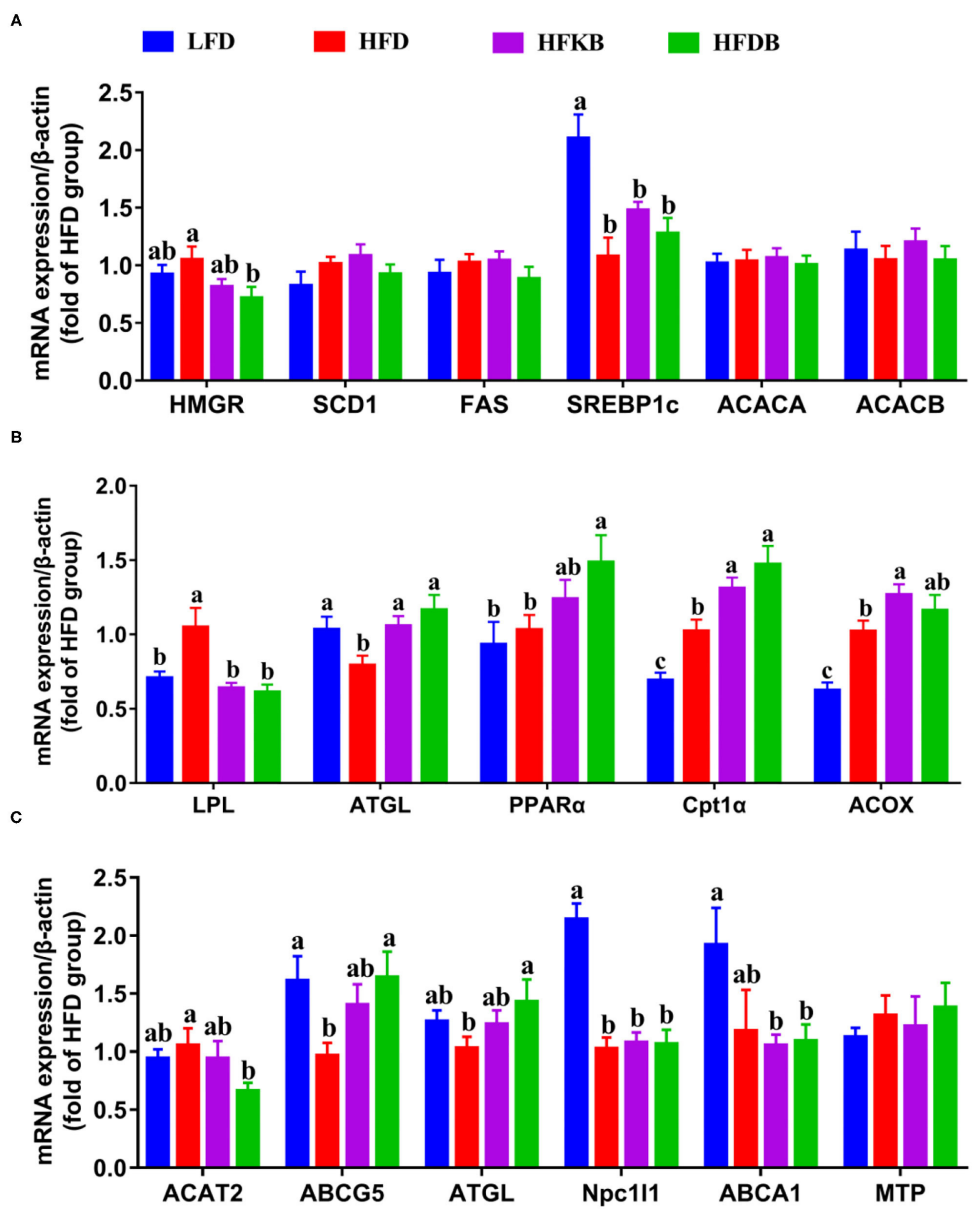
Effects of black tea on mRNA levels of genes in the liver and the small intestine. **(A,B)**, hepatic genes; **(C)** genes in the small intestine. Data are presented as mean ± SEM (*n* = 12). ^a,b,c^*p* represents significant differences among groups (ANOVA, *p* < 0.05).

To further examine the prevention of excessive fat deposition in the liver by dietary black tea, the expression of lipid-metabolizing genes in the liver was measured. The mRNA level of hepatic HMG-CoA reductase (HMGR) was significantly decreased by DBT, which neutralized the effects of the HFD treatment (Figure 4A). However, neither black tea treatment altered the mRNA expression of stearoyl-CoA desaturase 1 (SCD1), fatty acid synthase (FAS), sterol regulatory element binding protein-1c (SREBP1c), acetyl-CoA carboxylase A (ACACA), or acetyl-CoA carboxylase B (ACACB; Figure 4A). The expression of SCD1, FAS, ACACA, and ACACB among the four groups was comparable. Notably, the HFD treatment enhanced the mRNA level of lipoprotein lipase (LPL) and suppressed the expression of adipose triglyceride lipase (ATGL) in the liver; the black tea treatments completely reversed the HFD-induced changes of these two genes. The gene expression levels of peroxisome proliferator-activated receptor-alpha (PPARα), carnitine palmitoyl transterase-1 (Cpt1α), and acyl-CoA oxidase (ACOX) in mice undergoing black tea treatments were significantly enhanced relative to those of the HFD group (Figure 4B). Unlike the effects in alleviating fatty liver, the alterations of the mRNA expression of hepatic lipid–metabolizing genes by KBT or DBT treatment exhibited no significant differences.

### Effects of Dietary Black Tea on Expression of Genes Related to Lipid Transport and Metabolism in the Small Intestine

Given the notable effect of dietary black tea in promoting fecal lipid excretion, we quantified the mRNA expression levels of genes involved in lipid transport and metabolization in the small intestine. The HFD significantly increased the mRNA levels of acyl-CoA cholesterol acyltransferase 2 (ACAT2), Cpt1α, and ACOX and decreased the gene expression of ATP-binding cassette subfamily G 5 (ABCG5), ATGL, and Niemann–Pick C1-like 1 (Npc1l1; Figure 4C; Supplementary Figure 3A). Moreover, DBT treatment significantly decreased the gene expression of ACAT2 and increased the mRNA levels of ABCG5 and ATGL, indicating its neutralizing effects on the alterations resulting from high-fat feeding. The alterations of these genes through the KBT treatment exhibited similar trends; however, no significant differences were observed. Neither black tea treatment changed the gene expression of Npc1l1. Moreover, the mRNA levels of mitochondrial functional protein (MTP) and ATP-binding cassette transporter A1 among the four groups were comparable (Figure 4C). Similarly, neither black tea treatment altered the mRNA expression levels of genes involved in fatty acid re-esterification, prechylomicron assembly and secretion, or fatty acid metabolism in the small intestine (Supplementary Figures 2A–C).

### Effects of Dietary Black Tea on Gut Microbiota

**Figure 5:**
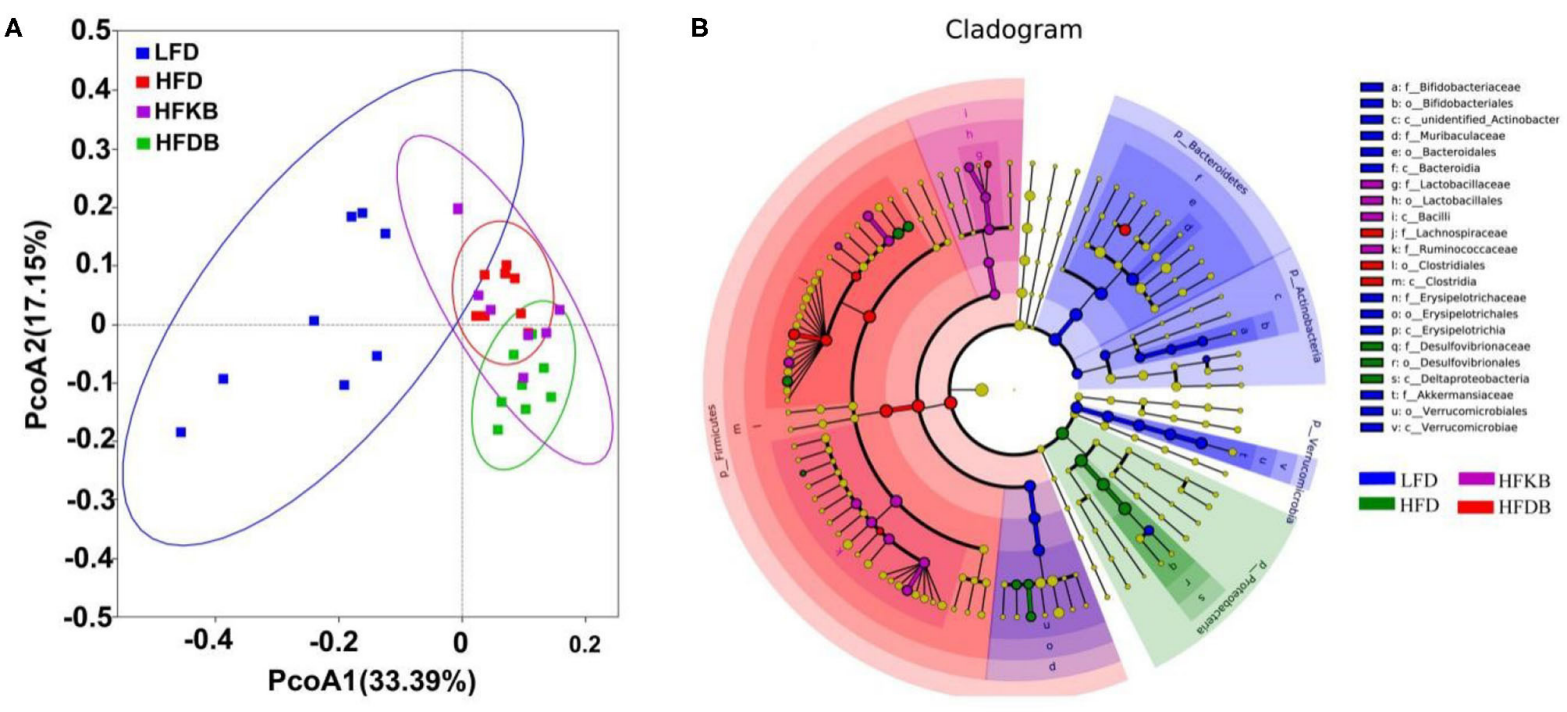
Effects of black tea on overall microbial structure. **(A)** principal coordinates analysis; **(B)** linear discriminant analysis effect size comparison of microbiota for LFD, HFD, HFKB, and HFDB.

That gut microbiota are closely related to HFD-induced obesity and non-alcoholic fatty liver disease (NAFLD) has been well documented (16). The concentration of fecal SCFAs was analyzed using gas chromatography, and the colonic microbiota were profiled using 16S rDNA gene sequencing. Our data indicated that total SCFA content, acetate, propionate, and butyrate were markedly decreased by high-fat feeding; however, neither black tea treatment prevented the changes induced by the feeding (Supplementary Table 4). Chao 1 and ACE estimators and the Shannon and Simpson indexes were used to assess community richness and diversity, respectively. No differences in Chao 1 and ACE estimations or the Shannon and Simpson indexes were evident among the four groups (Supplementary Table 5). However, the HFD induced a dramatic shift in gut microbiota; the proportion of Firmicutes increased and that of Bacteroidetes decreased, and the Firmicute to Bacteroidete ratio was significantly increased in HFD mice compared with the LFD animals. However, black tea treatment did not significantly affect the ratio (Supplementary Figure 3D). Similarly, in the results of principal coordinates analysis and linear discriminant analysis effect size, black tea supplementation exhibited little impact on the modulation of intestinal microbiota (Figure 5).

## Discussion

The health benefits of tea have become a hot topic in food science research (17). Black tea is the most consumed tea beverage worldwide. The major phenolic compounds of black tea are oxidized and dimerized to form theaflavins, which are of large molecular weight and not easily absorbed by the small intestine. The effects of black tea on fatty liver prevention in HFD-induced obese animals are well-established (18). Significant differences in chemical compositions are evident among categories of black tea; however, few studies have been conducted to systematically clarify the differences of chemical profiles and the potential health benefits of types of black tea. In the present study, two common categories of black tea in China were selected, and their chemical profiles and effects in alleviating excessive hepatic lipid depositions in mice fed an HFD were compared.

KBT and DBT are produced from a small-leaf tea plant cultivar (*C. sinensis* var. *sinensis*) and a large-leaf tea plant cultivar (*C. sinensis* var. *assamica*), respectively. In previous studies, the shoots and fresh leaves of large-leaf tea plant cultivars had higher contents of inclusion and more phenolic compounds than did small-leaf cultivars (19). Wang et al. (7) reported that tea polyphenols, flavonoids, and amino acids might play key roles in the differences between *C. sinensis* var. *sinensis* and *C. sinensis* var. *assamica*. In the present study, mass spectrographic analysis indicated that the marker compounds with the largest VIP values (≥2) between KBT and DBT were generally phenolic acids, catechins, and flavonoids, which is consistent with previous studies. Moreover, our former results (Supplementary Table 6) indicated that the amounts of total phenols, crude fiber, and tea polysaccharides in DBT were significantly higher than those in KBT, although the crude protein content did not differ. Therefore, in addition to small-molecule compounds (mainly phenolic compounds and theanine), tea polysaccharides and other macromolecular substances play vital roles in distinguishing between KBT and DBT.

Excessive fat consumption contributes to the development of obesity and NAFLD and causes hepatocellular damage and gut microbiota dysbiosis (20). The putative anti-obesity effects of tea have been most commonly ascribed to its rich bioactive compounds, especially the phenolic compounds (21). In the present study, HFD-treated mice exhibited the symptoms typical of obesity and NAFLD, and the results were consistent with those of other reports (20). Only dietary DBT significantly reduced body weight gain, perirenal adipose mass, serum ALT levels, and hepatic excess fat accumulation in mice fed an HFD, but these effects did not appear in the KBT group, with the exception of effects on perirenal adipose mass and serum ALT levels. Consistently, the significant alleviation of excessive fat accumulation and steatosis severity in the liver was observed only in the DBT-treated mice. We speculate that this phenomenon may be related to the differences in the levels of phenol compounds, theanine, and tea polysaccharides in the two types of tea.

Hepatic lipid metabolism disorder is a main cause of the development of fatty liver disease (22). We examined the expression of hepatic lipid–metabolizing genes. Both KBT and DBT significantly decreased the expression of hepatic HMGR, a rate-limiting enzyme in cholesterol biosynthesis (23), and the alteration of this gene might contribute to the decreased levels of hepatic cholesterol in black tea–treated mice. The excessive accumulation of hepatic cholesterol critically contributes to the pathogenesis of fatty liver (24). Our data demonstrated that dietary black tea was an effective approach to fatty liver prevention. Moreover, excess hepatic lipid accumulation usually reflects imbalances of fatty acid import and export, catabolism, and lipogenesis in the liver (25). In our study, the mRNA levels of genes involved in hepatic fatty acid *de novo* synthesis (FAS, ACACA, ACACB, and SCD1) were not significantly altered by black tea treatments. However, KBT and DBT significantly increased the mRNA expression of ATGL, PPARα, Cpt1α, and ACOX, which are related to fat lipolysis and fatty acid beta-oxidation. This result is consistent with a study in which Puerh tea treatment significantly increased mRNA levels of the transcription factor and enzymes involved in fatty acid oxidation, including PPARα, Cpt1α, and ACOX in HFD-induced obese mice (26). Notably, both black tea treatments completely restored the HFD-induced increase of LPL gene expression. LPL is an enzyme vital in catalyzing the hydrolysis of triglycerol from circulating chylomicrons or very low-density lipoproteins (27). The enhanced mRNA level of LPL in hepatic stellate cells increases the uptake of cholesterol from serum lipoproteins, which induces an increase in TLR4 signaling and the exacerbation of liver fibrosis (28). The decreased LPL mRNA levels evident with dietary black tea treatment indicated the role of LPL mRNA level in restraining the entry of free fatty acids and cholesterol into hepatic cells. Overall, the hepatic gene results suggested that dietary black tea might prevent the development of fatty liver by decreasing the synthesis of cholesterol, stimulating fat lipolysis and fatty acid oxidation, and inhibiting the absorption of cholesterol and free fatty acids from circulation. The alteration of hepatic lipid metabolism by KBT was comparable to that by DBT.

Our data also indicated that black tea treatments significantly increased fecal lipid excretion. We measured the expression of key genes related to the absorption, re-esterification, transport, and metabolism of lipid molecules in the small intestine. ACAT2 is a key enzyme for the re-esterification of cholesteryl ester, and the loss of ACAT2 results in defective cholesterol absorption (29). ABCG5 is a heterodimer involved in the transport of cholesterol from hepatocytes into the bile and from enterocytes into the intestinal lumen (30). Black tea treatments, especially DBT, significantly decreased the mRNA level of intestinal ACAT2 and increased the expression of ABCG5, which was notably suppressed by the HFD. DBT also effectively restored the reduced mRNA levels of intestinal ATGL in high-fat-feeding mice. These results implied that dietary DBT is more effective than dietary KBT in decreasing cholesterol absorption and promoting cholesterol efflux and lipolysis in the small intestine. However, the expression of most genes involved in the re-esterification, transport, and metabolism of intestinal lipid molecules was not altered by black tea supplementation. Our results indicated that black tea treatments did not alter the absorption of fat, especially the fatty acids that originate from neutral fat, which accounts for ~95% of dietary lipids. The inhibition of lipid digestion in the small intestine might be the main mechanism for the enhanced fecal fat excretion in black tea–treated mice. Previous studies have shown that dietary tea powder, tea polyphenols, and epigallocatechin gallate can decrease the activity of pancreatic lipase and alter the emulsification of dietary lipids (31). Therefore, black tea supplementation significantly stimulated fecal fat excretion but did not alter the absorption of lipid molecules in the small intestine. The stimulatory effects of DBT on fecal lipid excretion were significantly stronger than those of KBT, and this might be the primary contributor to the differences between the two black teas in preventing the development of fatty liver.

In the past decade, the relationship between the development of fatty liver and the alteration of the gut microbiota has been frequently reported in several lines of studies (32). As the metabolites of microorganisms, the composition and amounts of fecal SCFAs can reveal the status of the gut microbiota (33). Our data demonstrated that the HFD significantly decreased the amount of intestinal SCFAs, and black tea supplementation did not recover the production of fecal SCFAs. Similarly, the amelioration of the Firmicutes to Bacteroidetes ratio was not observed in black tea–treated mice. Furthermore, black tea supplementation did not change the α- and β-diversity of intestinal microbiota, indicating that intestinal microbes made no significant contribution to the improvement of fatty liver observed in our study. The reason for these intricate results may be related to the cycle of the high-fat model and the dosage of the black teas.

Our results demonstrated that DBT was more effective than KBT in alleviating excessive hepatic lipid accumulation in mice fed an HFD. In UPLC-Q-TOF-MS/MS data, the compounds crucial in differentiating KBT and DBT were quinic acid, kaempferol-3-O-glucoside or its isomer, theanine, isoquercitrin or its isomer, and others, and these may be key factors in the differences in the health benefits of the teas. Quinic acid and chlorogenic acid are phenolic acids, and either can effectively prevent fatty liver disease through the alteration of fat metabolism in the liver and other organs (34). Kaempferol-3-O-glucoside, isoquercitrin, rutin, and hyperoside are active flavonoids in black tea and can prevent fatty liver by inhibiting lipogenesis and facilitating fatty acid metabolism (35). Theanine and L-glutamine are amino acids that effectively ameliorated lipid metabolism disorders in male Sprague–Dawley rats and C57BL/6J mice (36). D-psicose is a new-generation sugar substitute that suppressed lipogenesis and stimulated fatty acid oxidation in Wistar rats (37). As one of the widely studied active compounds in black tea, phenolic acids are considered to be the crucial compounds of health benefits, including catechins (38), gallic acid (39) and other compounds found in black tea, is likely to be the most crucial compounds responsible for the stronger beneficial effects of DBT than KBT in the prevention of fatty liver, which is a limitation that need to be addressed in additional studies. Besides, tea polysaccharide is also an important functional ingredient in black tea, and whether the content and structure of tea polysaccharide play an important role in promoting the gut health warrants further studied. Collectively, these results indicate that most of the compounds crucial to the differentiation of the KBT and DBT samples play vital roles in the prevention of fatty liver. However, the compound or combination of compounds that is the key factor in the differences in the health benefits of the two black teas is not yet clear.

## Conclusion

In conclusion, most of the compounds differentiating KBT and DBT are phenolic compounds, theanine, and D-psicose. DBT was more effective than KBT in preventing excess fat accumulation in the liver of mice fed an HFD. Both black tea treatments effectively and comparably altered the mRNA levels of hepatic genes involved in cholesterol synthesis, fat lipolysis, fatty acid beta-oxidation, and the absorption of free fatty acid and cholesterol from circulation. DBT treatment exhibited more favorable effects in stimulating fecal fat excretion than did KBT treatment, and this may be the primary factor in the different health-promoting effects of the two tea treatments in this study. The different compounds with the higher VIP values might make the main contributions to the different health benefits; however, the most important compound or combination of compounds requires clarification.

## Data Availability Statement

The data presented in the study are deposited in the NCBI Trace Archive NCBI Sequence Read Archive repository, accession number PRJNA804701.

## Ethics Statement

The animal study was reviewed and approved by the Institutional Animal Care and Use Committee of Anhui Agricultural University.

## Author Contributions

WL: investigation, methodology, data curation, and writing - original draft. SL: investigation, methodology, and data curation. YC and YK: investigation and methodology. DW and TL: methodology and writing-review and editing. YW and IK: writing-review and editing. ZX: funding acquisition and writing-review and editing. JH: conceptualization, supervision, funding acquisition, and writing-review and editing. All authors contributed to the article and approved the submitted version.

## Funding

This work was supported by the Key Research and Development Program of Anhui Province (Grant Number 201904b11020038), the Natural Science Foundation of Anhui province, China (Grant Number 2108085MC119), the National Natural Science Foundation (Grant Number 31972459), and a Key Joint Grant for Regional Innovation and Development from National Sciences Foundation of China (Grant Number U19A2034).

## Conflict of Interest

The authors declare that the research was conducted in the absence of any commercial or financial relationships that could be construed as a potential conflict of interest.

## Publisher's Note

All claims expressed in this article are solely those of the authors and do not necessarily represent those of their affiliated organizations, or those of the publisher, the editors and the reviewers. Any product that may be evaluated in this article, or claim that may be made by its manufacturer, is not guaranteed or endorsed by the publisher.

## References

[1] Li X Wang H Wang T Zheng F Wang C. Dietary wood pulp-derived sterols modulation of cholesterol metabolism and gut microbiota in high-fat-diet-fed hamsters. Food Funct. (2019) 10:775–85. 10.1039/C8FO02271B 30667436

[2] Rouabhia S Milic N Abenavoli L. Metformin in the treatment of non-alcoholic fatty liver disease: safety, efficacy and mechanism. Expert Rev Gastroenterol Hepatol. (2014) 8:343–9. 10.1586/17474124.2014.894880 24580044

[3] Salomone F Godos J Zelber-Sagi S. Natural antioxidants for non-alcoholic fatty liver disease: molecular targets and clinical perspectives. Liver Int. (2016) 36:5–20. 10.1111/liv.12975 26436447

[4] Yang CS Zhang J Zhang L Huang J Wang Y. Mechanisms of body weight reduction and metabolic syndrome alleviation by tea. Mol Nutr Food Res. (2016) 60:160–74. 10.1002/mnfr.201500428 26577614 PMC4991829

[5] Chang CW Wang SH Jan MY Wang WK. Effect of black tea consumption on radial blood pulse spectrum and cognitive health. Complement Ther Med. (2017) 31:1–7. 10.1016/j.ctim.2017.01.001 28434461

[6] Gao Y Xu Y Ruan J Yin J. Selenium affects the activity of black tea in preventing metabolic syndrome in high-fat diet-fed Sprague-Dawley rats. J Sci Food Agric. (2020) 100:225–34. 10.1002/jsfa.10027 31512247

[7] Wang CB Lyu H Guo ZY. Metabolomic and pathway changes in large-leaf, middle-leaf and small-leaf cultivars of *Camellia sinensis* (L.) Kuntze var. niaowangensis. Chem Biodivers. (2021) 18:e2100132. 10.1002/cbdv.202100132 33928738

[8] Wei CL Yang H Wang SB Zhao J Liu C Gao LP. Draft genome sequence of *Camellia sinensis* var. sinensis provides insights into the evolution of the tea genome and tea quality. Proc Natl Acad Sci USA. (2018) 115:E4151–E4158. 10.1073/pnas.1719622115 29678829 PMC5939082

[9] Guo X Long P Meng Q Ho CT Zhang L. An emerging strategy for evaluating the grades of Keemun black tea by combinatory liquid chromatography-Orbitrap mass spectrometry-based untargeted metabolomics and inhibition effects on alpha-glucosidase and alpha-amylase. Food Chem. (2018) 246:74–81. 10.1016/j.foodchem.2017.10.148 29291881

[10] Zhu J Zhu F Li L Cheng L Zhang L Sun Y. Highly discriminant rate of Dianhong black tea grades based on fluorescent probes combined with chemometric methods. Food Chem. (2019) 298:125046. 10.1016/j.foodchem.2019.125046 31260981

[11] Zhou J Wu Y Long P Ho CT Wang Y Kan Z. LC-MS-based metabolomics reveals the chemical changes of polyphenols during high-temperature roasting of large-leaf yellow tea. J Agric Food Chem. (2019) 67:5405–5412. 10.1021/acs.jafc.8b05062 30485095

[12] Huang J Feng S Liu A Dai Z Wang H Reuhl K. Green tea polyphenol EGCG alleviates metabolic abnormality and fatty liver by decreasing bile acid and lipid absorption in mice. Mol Nutr Food Res. (2018) 62:1700696. 10.1002/mnfr.201700696 29278293 PMC6350933

[13] Kim I Ahn SH Inagaki T Choi M Ito S Guo GL. Differential regulation of bile acid homeostasis by the farnesoid X receptor in liver and intestine. J Lipid Res. (2007) 48:2664–72. 10.1194/jlr.M700330-JLR200 17720959

[14] Tian L Scholte J Borewicz K van den Bogert B Smidt H Scheurink AJ. Effects of pectin supplementation on the fermentation patterns of different structural carbohydrates in rats. Mol Nutr Food Res. (2016) 60:2256–66. 10.1002/mnfr.201600149 27174558

[15] Edgar RC. UPARSE: highly accurate OTU sequences from microbial amplicon reads. Nat Methods. (2013) 10:996–8. 10.1038/nmeth.2604 23955772

[16] Liu J Hao W He Z Kwek E Zhao Y Zhu H. Beneficial effects of tea water extracts on the body weight and gut microbiota in C57BL/6J mice fed with a high-fat diet. Food Funct. (2019) 10:2847–60. 10.1039/C8FO02051E 31062778

[17] Shang A Li J Zhou DD Gan RY Li HB. Molecular mechanisms underlying health benefits of tea compounds. Free Radic Biol Med. (2021) 172:181–200. 10.1016/j.freeradbiomed.2021.06.006 34118386

[18] Xu J Li M Zhang Y Chu S Huo Y Zhao J. Huangjinya black tea alleviates obesity and insulin resistance via modulating fecal metabolome in high-fat diet-fed mice. Mol Nutr Food Res. (2020) 64:e2000353. 10.1002/mnfr.202000353 33002297

[19] Yang T Xie Y Lu X Yan X Wang Y Ma J. Shading promoted theanine biosynthesis in the roots and allocation in the shoots of the tea plant (*Camellia sinensis* L.) cultivar shuchazao. J Agric Food Chem. (2021) 69:4795–803. 10.1021/acs.jafc.1c00641 33861578

[20] Chiu S Mulligan K Schwarz JM. Dietary carbohydrates and fatty liver disease: de novo lipogenesis. Curr Opin Clin Nutr Metab Care. (2018) 21:277–82. 10.1097/MCO.0000000000000469 29697539

[21] Rains TM Agarwal S Maki KC. Antiobesity effects of green tea catechins: a mechanistic review. J Nutr Biochem. (2011) 22:1–7. 10.1016/j.jnutbio.2010.06.006 21115335

[22] Neuschwander-Tetri BA. Hepatic lipotoxicity and the pathogenesis of nonalcoholic steatohepatitis: the central role of nontriglyceride fatty acid metabolites. Hepatology. (2010) 52:774–88. 10.1002/hep.23719 20683968

[23] Liu W Zhang Z Li W Zhu W Ren Z Wang Z. Genome-wide identification and comparative analysis of the 3-hydroxy-3-methylglutaryl coenzyme a reductase (HMGR) gene family in gossypium. Molecules. (2018) 23:193. 10.3390/molecules23020193 29364830 PMC6017885

[24] Arguello G Balboa E Arrese M Zanlungo S. Recent insights on the role of cholesterol in non-alcoholic fatty liver disease. Biochim Biophys Acta. (2015) 1852:1765–78. 10.1016/j.bbadis.2015.05.015 26027904

[25] Tolman KG Dalpiaz AS. Treatment of non-alcoholic fatty liver disease. Ther Clin Risk Manag. (2007) 3:1153–63. 10.1159/000447278 18516264 PMC2387293

[26] Huang F Wang S Zhao A Zheng X Zhang Y Lei S. Pu-erh tea regulates fatty acid metabolism in mice under high-fat diet. Front Pharmacol. (2019) 10:63. 10.3389/fphar.2019.00063 30804786 PMC6370627

[27] He PP Jiang T OuYang XP Liang YQ Zou JQ Wang Y. Lipoprotein lipase: biosynthesis, regulatory factors, and its role in atherosclerosis and other diseases. Clin Chim Acta. (2018) 480:126–37. 10.1016/j.cca.2018.02.006 29453968

[28] Teratani T Tomita K Furuhashi H Sugihara N Higashiyama M Nishikawa M. Lipoprotein lipase up-regulation in hepatic stellate cells exacerbates liver fibrosis in nonalcoholic steatohepatitis in mice. Hepatol Commun. (2019) 3:1098–112. 10.1002/hep4.1383 31388630 PMC6671781

[29] Nguyen TM Sawyer JK Kelley KL Davis MA Rudel LL. Cholesterol esterification by ACAT2 is essential for efficient intestinal cholesterol absorption: evidence from thoracic lymph duct cannulation. J Lipid Res. (2012) 53:95–104. 10.1194/jlr.M018820 22045928 PMC3243485

[30] Graf GA Yu L Li WP Gerard R Tuma PL Cohen JC. ABCG5 and ABCG8 are obligate heterodimers for protein trafficking and biliary cholesterol excretion. J Biol Chem. (2003) 278:48275–82. 10.1074/jbc.M310223200 14504269

[31] Seo DB Jeong HW Kim YJ Kim S Kim J Lee JH. Fermented green tea extract exhibits hypolipidaemic effects through the inhibition of pancreatic lipase and promotion of energy expenditure. Br J Nutr. (2017) 117:177–86. 10.1017/S0007114516004621 28132656

[32] Ushiroda C Naito Y Takagi T Uchiyama K Mizushima K Higashimura Y. Green tea polyphenol (epigallocatechin-3-gallate) improves gut dysbiosis and serum bile acids dysregulation in high-fat diet-fed mice. J Clin Biochem Nutr. (2019) 65:34–46. 10.3164/jcbn.18-116 31379412 PMC6667385

[33] Liu B Wang W Zhu X Sun X Xiao J Li D. Response of gut microbiota to dietary fiber and metabolic interaction with SCFAs in piglets. Front Microbiol. (2018) 9:2344. 10.3389/fmicb.2018.02344 30323803 PMC6172335

[34] Xie M Chen G Wan P Dai Z Zeng X Sun Y. Effects of dicaffeoylquinic acids from Ilex kudingcha on lipid metabolism and intestinal microbiota in high-fat-diet-fed mice. J Agric Food Chem. (2019) 67:171–83. 10.1021/acs.jafc.8b05444 30561211

[35] Qin G Ma J Huang Q Yin H Han J Li M. Isoquercetin improves hepatic lipid accumulation by activating AMPK pathway and suppressing TGF-beta signaling on an HFD-induced nonalcoholic fatty liver disease rat model. Int J Mol Sci. (2018) 19:4126. 10.3390/ijms19124126 30572631 PMC6321444

[36] Lin L Zeng L Liu A Peng YQ Yuan DY Zhang S. l-Theanine regulates glucose, lipid, and protein metabolism via insulin and AMP-activated protein kinase signaling pathways. Food Funct. (2020) 11:1798–809. 10.1039/C9FO02451D 32057039

[37] Chen J Huang W Zhang T Lu M Jiang B. Anti-obesity potential of rare sugar d-psicose by regulating lipid metabolism in rats. Food Funct. (2019) 10:2417–25. 10.1039/C8FO01089G 30964474

[38] Xie J Li J Liang J Luo P Qing LS Ding LS. Determination of contents of catechins in oolong teas by quantitative analysis of multi-components via a single marker (QAMS) method. Food Anal Method. (2017) 10:363–8. 10.1007/s12161-016-0592-5

[39] Tung YT Huang CZ Lin JH Yen GC. Effect of *Phyllanthus emblica* L. fruit on methionine and choline-deficiency diet-induced nonalcoholic steatohepatitis. J Food Drug Anal. (2018) 26:1245–52. 10.1016/j.jfda.2017.12.005 30249323 PMC9298569

