# Supplementary material for: Effects of Keemun and Dianhong Black Tea in Alleviating Excess Lipid Accumulation in the Liver of Obese Mice: A Comparative Study
Source: Front Nutr. 2022 Mar 15;9:849582. doi: 10.3389/fnut.2022.849582 (PMC8967360; doi:10.3389/fnut.2022.849582)
Supplement: Supplementary file 1 [file Data_Sheet_1.docx]

**Table S1. Ingredient composition of the high fat diet**

| Ingredients | Contents (g/kg) |
| --- | --- |
| Casein | 257 |
| Corn Starch | 0 |
| Maltodextrin | 161 |
| Sucrose | 88 |
| Soybean Oil | 32 |
| Lard | 315 |
| Cellulose | 64 |
| Mineral Mix | 63 |
| Vitamin Mix | 13 |
| L-Cystine | 4 |
| Choline Bitartrate | 3 |
| TBHQ | 0.069 |
| Total | 1000 |

**Table S2.** **Primers used for real-time PCR**

| **Gene Name** | **Gene ID** | **Primer Sequence** |
| --- | --- | --- |
| ACOX | 11430 | F:5'-GCTGTTAAGAAGAGTGCCACC -3' |
|  |  | R:5'-GTGCATCCATTTCTCCTGCTG -3' |
| FAS | 14102 | F:5'-GCTGCGGAAACTTCAGGAAAT -3' |
|  |  | R:5'-AGAGACGTGTCACTCCTGGACTT -3' |
| CD36 | 12491 | F:5'-GATGACGTGGCAAAGAACAG -3' |
|  |  | R:5'-TCCTCGGGGTCCTGAGTTAT -3' |
| HMGR | 15357 | F:5'-CTTGTGGAATGCCTTGTGATTG-3' |
|  |  | R:5'-AGCCGAAGCAGCACATGAT-3' |
| CYP7A1 | 13122 | F:5'-AACAACCTGCCAGTACTAGATAGC -3' |
| LPL | 16956 | R:5'-GTGTAGAGTGAAGTCCTCCTTAGC -3' |
|  |  | R: 5'-CCTGCTGTCTTCTAATGCTG -3' |
| SR-BI | 20778 | F:5'-TCCCCATGAACTGTTCTGTGAA-3' |
|  |  | R:5'-TGCCCGATGCCCTTGACA-3' |
| LDLR | 16835 | F:5'-AGGCTGTGGGCTCCATAGG-3' |
|  |  | R:5'-TGCGGTCCAGGGTCATCT-3' |
| FATP4 | 26569 | F:5'- AGCTGCCTCTGTATGCC -3  R:5'- ACAGCGGGTCTTTCACAAC -3' |
| FABP1 | 14080 | F: 5'- ACCTATGGACCCAAAGTGGTC-3'  R: 5'- CTTGACGACTGCCTTGACTTT-3' |
| FABP2 | 14079 | F:5'- ATGCCCACATGCTGTAGTTGA -3'  R:5'- TAACCTAACCGCCTCACATGC -3' |
| Apoa4 | 11808 | F:5'- CGTGCAGGAGAAACTCAACC-3'  R:5'- GTTGCCCTTCACCTTGCT -3' |
| ApoE | 11816 | F:5'- CAAGAACTGACGGCACT-3'  R:5'- GGACCCAGCTGTTCCT -3' |
| ApoB | 238055 | F:5'- GGAAGCTGTTGCTGAGTGGT -3'  R:5'- ACCCTTCTCTGATGGCCTGT -3' |
| MGAT2 | 233549 | F:5'- ATGGGAGCGCAGGTTACAGAC-3'  R:5'- GGCCTACGAAGATGACGATGC-3' |
| DGAT1 | 13350 | F:5'- CGCTTTCTGCTACGACGA -3'  R:5'- AGACGCTCAATGATCCG -3' |
| DGAT2 | 67800 | F:5'- ACTCTGGAGGTTGGCACCAT-3'  R:5'- GGGTGTGGCTCAGGAGGAT-3' |
| CGI-58 | 67469 | F:5'- CCTGTCTATGCCTTTGACC-3'  R:5'- TTCTCCACTTCTTCCGCATC-3' |
| ABCG5 | 27409 | F:5'- TGGATCCAACACCTCTATGCTAAA-3'  R:5'- GGCAGGTTTTCTCGATGAACTG-3' |
| ABCA1 | 11303 | F:5'- CGTTTCCGGGAAGTGTCCTA-3'  R:5'- GCTAGAGATGACAAGGAGGATGGA-3' |
| Npc1l1 | 237636 | F:5'- ATGAACGCCATTTGCTCT-3'  R:5'- GCAATAGCCACATAAGACTG-3' |
| ACAT2 | 110460 | F: 5'- TGCCTGTCTCTCGGTTT-3'  R: 5'- AACAATCCAGAGGCACAC-3' |
| Plin2 | 11520 | F:5'- TGGCTGTAAACGTCTGTCTGG-3'  R:5'- CACACGCCTTGAGAGAAACAG-3' |
| Plin3 | 66905 | F: 5'- TATGAACACTCCCTCGGCAAG-3'  R: 5'- GTTTCACGGATTCCATCAGGC -3' |
| Sar1b | 66397 | F: 5'- ATGAAACCATTGCCAACGTGC-3'  R: 5'- CACATGAACACTTCCAGAGGC-3' |
| HNF4α | 15378 | F: 5'- GCCCCTGCAAAGTGTCA-3'  R: 5'- CTCACAGCCCATTCCT-3' |
| MTP | 17777 | F: 5'- TGAATTAAAAGGCCACACCAA-3'  R: 5'- CTCAGCTAAACGTCCACT-3' |
| PPARα | 19013 | F:5'-TCATCAAGAAGACCGAGTCC-3' |
|  |  | R:5'-CCTCTTCATCCCCAAGCGTA-3' |
| SCD1 | 20249 | F:5'-TCCTCCTTGGATTGTGTAGAAACTT-3' |
|  |  | R:5'-AATGTCAGAAGAAATCAGGTGGGTA-3' |
| ACACB | 100705 | F: 5'-AGACACTGCAAATCCCAACCTTAC-3' |
|  |  | R: 5'-CTTCGTCCACATCCTTCACACA-3' |
| LXRα | 22259 | F:5'-TCAGAAGAACAGATCCGCTTG-3' |
|  |  | R:5'-CGCCTGTTACACTGTTGCT-3' |
| ACACA | 107476 | F: 5'- AGGAGGGAAAGGGATCAGAAAAG-3' |
|  |  | R: 5'- CAGAGCAGTCACGACCAAACAAA -3' |
| SREBP1c | 20787 | F: 5'- AGTCCAGCCTTTGAGGATAGCC-3' |
|  |  | R: 5'- CCGTAGCATCAGAGGGAGTGAG-3' |
| Cpt1α | 12894 | F: 5'- CATCCACGCCATACTGCT-3' |
|  |  | R: 5'- GACCTTGAAGTAACGGCCTC-3' |
| ATGL | 66853 | F: 5'- CACCAGCATCCAGTTCAACCT-3' |
|  |  | R: 5'- AAGGGTTGGGTTGGTTCAGTA-3' |
| β-Actin | 11461 | F: 5'-TCCGGACATTCAACCATCAC-3' |
|  |  | R: 5'-TCACTGCACATCCCAGATCTC-3' |

**Table S3. Serum lipid indexes of the mice**

|  | LFD | HFD | HFKB | HFDB |
| --- | --- | --- | --- | --- |
| TG (mmol/L) | 0.68 ± 0.30 | 0.64 ± 0.10 | 0.53 ± 0.16 | 0.61 ± 0.19 |
| TC (mmol/L) | 2.54 ± 0.84^b^ | 5.84 ± 1.35^a^ | 5.84 ± 0.91^a^ | 5.71 ± 1.60^a^ |
| HDL-C (mmol/L) | 3.06 ± 1.47^b^ | 5.11 ± 1.97^a^ | 4.71 ± 1.38^a^ | 4.90 ± 1.51^a^ |
| LDL-C (mmol/L) | 0.63 ± 0.45^b^ | 2.17 ± 1.22^a^ | 3.54 ± 1.51^a^ | 2.67 ± 1.90^a^ |

Note: TG, triacylglycerol; TC, total cholesterol; HDL-C, high density lipoprotein cholesterol; LDL-C, low density lipoprotein cholesterol. Values are shown as mean ± SEM (n = 12). Different letters indicate significant differences (ANOVA, p<0.05).

**Table S4. Concentration of short-chain fatty acids (SCFAs) in per gram fecal (μmol/g)**

| SCFAs | LFD | HFD | HFKB | HFDB |
| --- | --- | --- | --- | --- |
| Acetate | 36.45 ± 6.40^a^ | 19.06 ± 1.53^b^ | 17.62 ± 1.16^b^ | 17.50 ± 2.38^b^ |
| Propionate | 2.41 ± 0.56^a^ | 1.23 ± 0.35^b^ | 0.99 ± 0.06^b^ | 1.02 ± 0.17^b^ |
| Butyrate | 6.47 ± 0.17^a^ | 0.50 ± 0.04^b^ | 0.48 ± 0.03^b^ | 0.50 ± 0.02^b^ |
| Total SCFAs | 45.32 ± 7.08^a^ | 20.80 ± 1.88^b^ | 19.09 ± 1.13^b^ | 19.02 ± 2.52^b^ |

Note: Samples were collected on week 15. Data are presented as the mean ± standard error of the mean (n = 8). Different letters indicate significant difference values (ANOVA, p<0.05).

**Table S5.** **Richness and α-diversity of mice in different experimental groups**

|  | **Chao1** | **ACE** | **Shannon** | **Simpson** |
| --- | --- | --- | --- | --- |
| **LFD** | 357.8 ± 12.1 | 355.8 ± 12.0 | 4.90 ± 0.34 | 0.910 ± 0.033 |
| **HFD** | 340.3 ± 8.8 | 340.3 ± 10.4 | 4.77 ± 0.25 | 0.874 ± 0.024 |
| **HFKB** | 361.6 ± 49.7 | 361.4 ± 50.3 | 5.04 ± 0.42 | 0.885 ± 0.023 |
| **HFDB** | 344.3 ± 5.2 | 344.2 ± 4.5 | 5.19 ± 0.19 | 0.912 ± 0.021 |

Note: Data are presented as the mean ± standard error of the mean (n = 8). Different letters indicate significant difference values (ANOVA, p<0.05).**Table S6.*** **Comparison of compounds of Keemun black tea and Dianhong black tea**

| Compound | Keemun Black Tea | Dianhong BlackTea |
| --- | --- | --- |
| TPC | 79.35 ± 2.82^b^ | 126.95 ± 1.30^a^ |
| EGC | 1.30 ± 0.06^b^ | 33.92 ± 0.60^a^ |
| C | ND | 1.18 ±0.12 |
| EC | 0.60 ± 0.03^b^ | 2.56 ± 0.10^a^ |
| EGCG | 3.14 ± 0.06^b^ | 8.65 ± 0.62^a^ |
| ECG | 1.68 ± 0.07^b^ | 17.61 ± 0.69^a^ |
| THB | 1.05 ± 0.04^b^ | 4.72 ± 0.10^a^ |
| CAF | 47.53 ± 1.02^b^ | 53.24 ± 1.56^a^ |
| Theanine | 12.99 ± 0.52^a^ | 4.40 ± 0.42^b^ |
| TF | 1.19 ± 0.01^b^ | 1.34 ± 0.01^a^ |
| TF3G | 7.78 ± 0.11^a^ | 6.35 ± 0.13^b^ |
| TF3'G | 2.48 ± 0.05^b^ | 3.23 ± 0.07^a^ |
| TFDG | 24.58 ± 0.41^a^ | 19.02 ± 0.37^b^ |
| GA | 3.14 ± 0.06^b^ | 4.66 ± 0.14^a^ |
| TPS | 25.33 ± 0.12^b^ | 47.82 ± 5.10^a^ |
| CPC | 343.43 ± 1.62^a^ | 256.63 ± 5.68^b^ |
| CFC | 109.55 ± 0.95^b^ | 121.65 ± 1.45^a^ |

Note: Values are means ± SEM (n=3). TPC: total phenol content; EGC: (−)-epigallocatechin; C: (+)-catechin; EC: (−)-epicatechin; EGCG: (−)-epigallocatechin gallate; ECG: (−)-epicatechingallate; THB: theobromine; CAF: caffeine. TF: Theaflavin; TF3G: Theaflavin-3-monogallate; TF3'G: Theaflavin-3’-monogallate; TFDG: Theaflavin-3,3’-digallate; GA: gallic acid; TPS: Tea polysaccharide; CPC: Crude protein content; CFC: Crude fiber content. ND: not detect. Different letters indicate significant differences (p<0.05).

* These data were published in our previous studies.

**
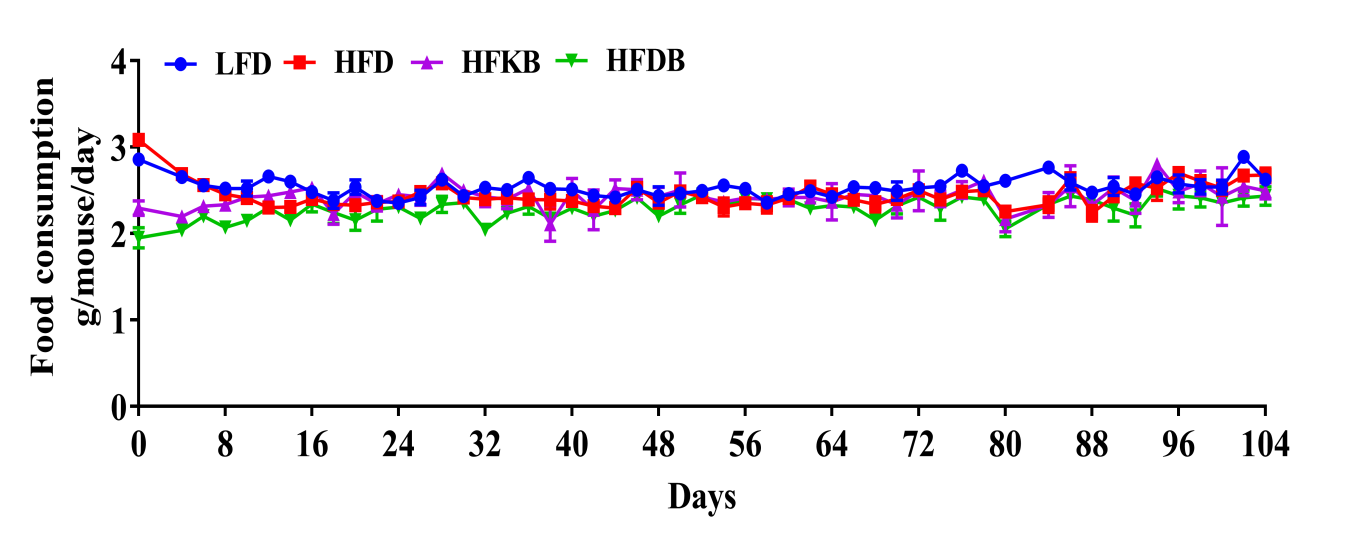
**

**Figure S1. Effects of black tea on food consumption.** All data are expressed as mean ± SEM (n = 12).

**
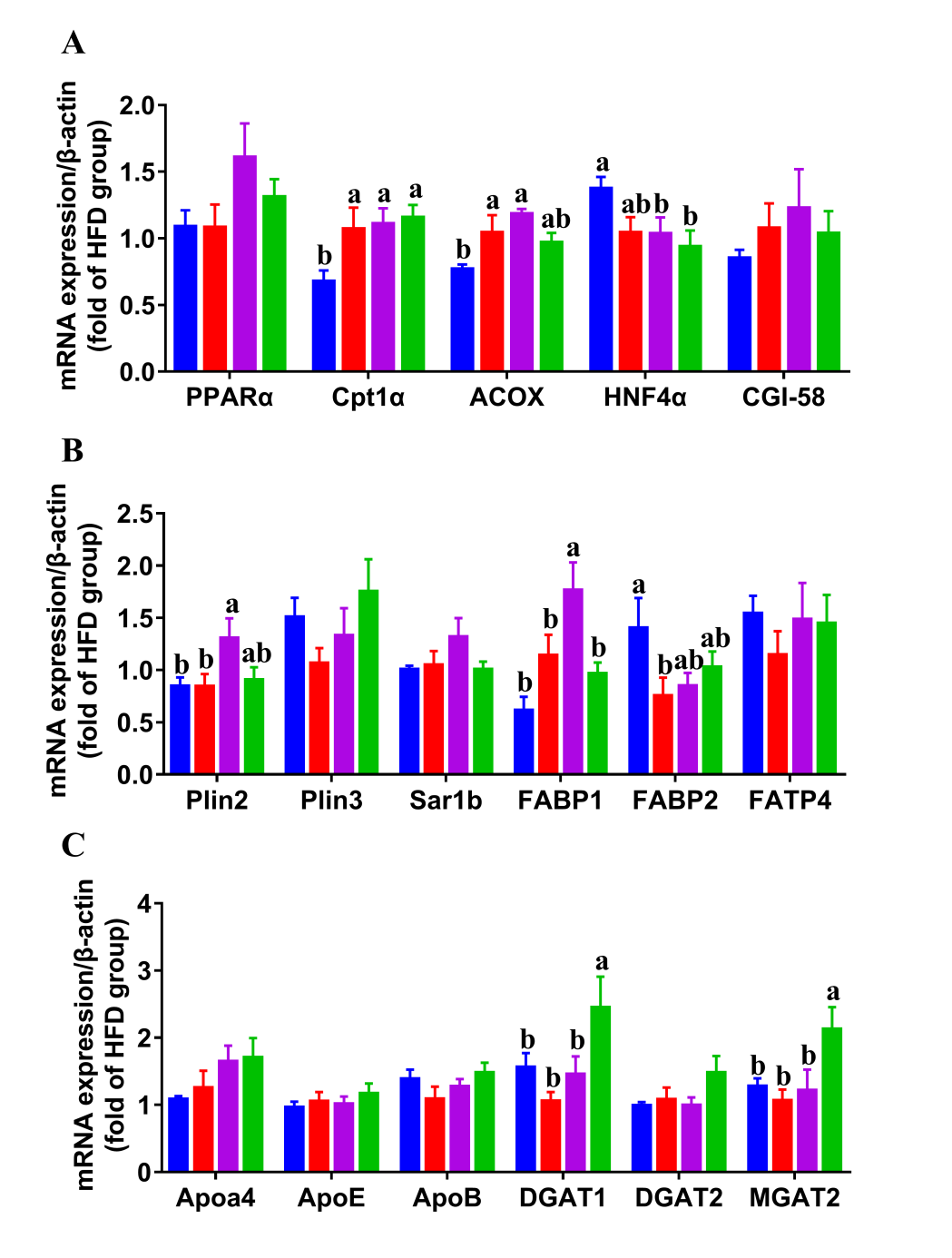
**

**Figure S2. Effects of black tea on mRNA levels of key genes related to lipid metabolism and transport in the small intestine.** All data are expressed as mean ± SEM (n = 12). Letters indicate significance of difference (ANOVA, *p* < 0.05).

**
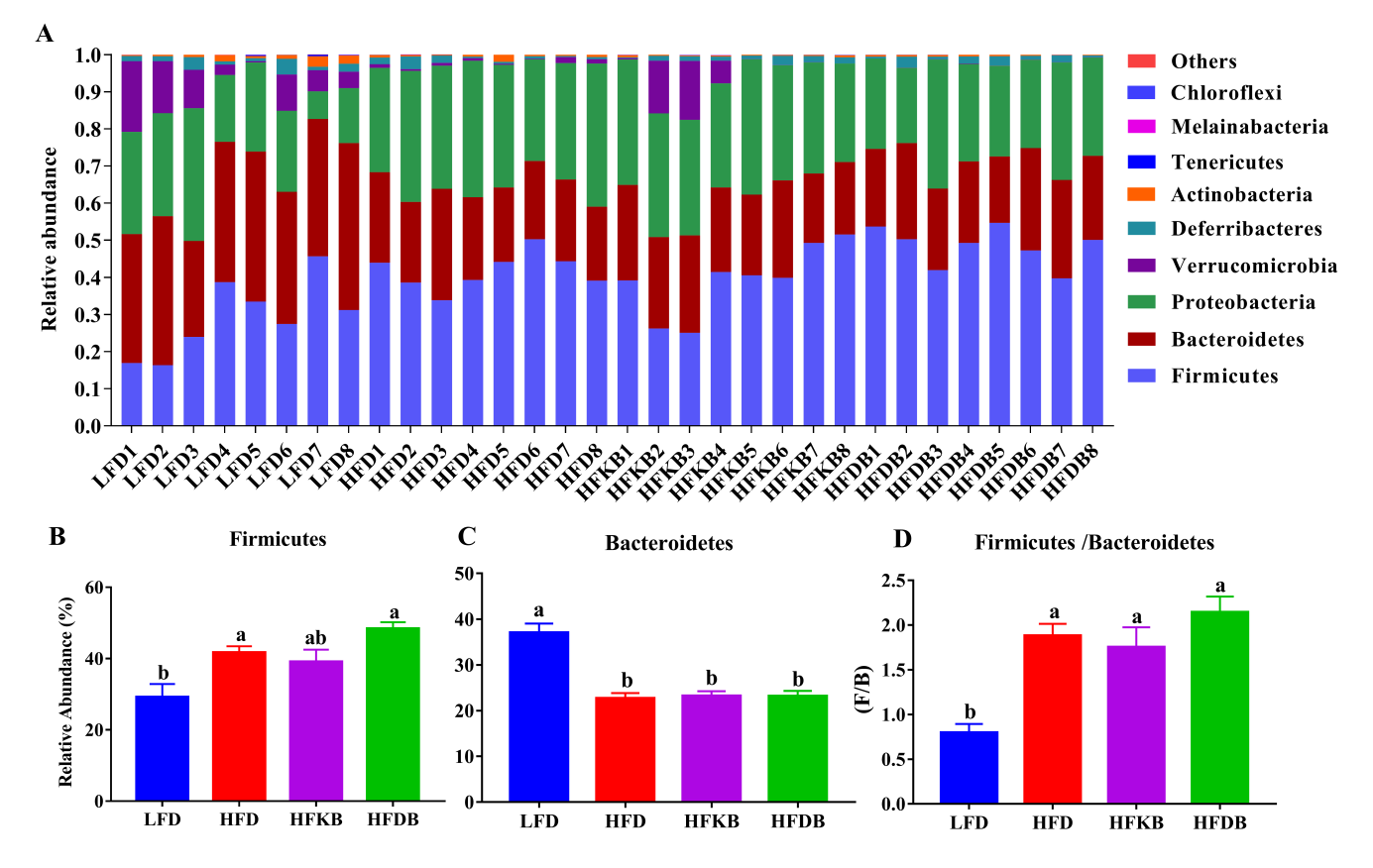
**

**Figure S3. Effects of black tea on relative abundance of fecal microbiota at the phylum level.** All data are expressed as mean ± SEM (n = 8). Letters indicate significance of difference (ANOVA, *p* < 0.05).
